# The combination of high-fat diet-induced obesity and chronic ulcerative colitis reciprocally exacerbates adipose tissue and colon inflammation

**DOI:** 10.1186/1476-511X-10-204

**Published:** 2011-11-10

**Authors:** Lílian G Teixeira, Alda J Leonel, Edenil C Aguilar, Nathália V Batista, Andréa C Alves, Candido C Coimbra, Adaliene VM Ferreira, Ana Maria C de Faria, Denise C Cara, Jacqueline I Alvarez Leite

**Affiliations:** 1 Department of Biochemistry and Immunology - Institute of Biological Sciences -Universidade Federal de Minas Gerais, Av. Antonio Carlos, 6627, Pampulha, Belo Horizonte, MG CEP: 31270-901, Brazil; 2 Department of Morphology - Institute of Biological Sciences - Universidade Federal de Minas Gerais, Av. Antonio Carlos, 6627, Pampulha, Belo Horizonte, MG CEP: 31270-901, Brazil; 3 Department of Physiology and Biophysics - - Institute of Biological Sciences - Universidade Federal de Minas Gerais, Av. Antonio Carlos, 6627, Pampulha, Belo Horizonte, MG CEP: 31270-901, Brazil; 4 Department of Basic Nursing - School of Nutrition - Universidade Federal de Minas Gerais, Av. Alfredo Balena, 190, Santa Efigênia, Belo Horizonte, MG CEP: 30130-100, Brazil

**Keywords:** ulcerative colitis, obesity, high-fat diet-induced obesity and inflammation

## Abstract

**Background:**

This study evaluated the relationship between ulcerative colitis and obesity, which are both chronic diseases characterized by inflammation and increases in immune cells and pro-inflammatory cytokines.

**Methods:**

Mice with chronic ulcerative colitis induced by 2 cycles of dextran sodium sulfate (DSS) in the first and fourth week of the experiment were fed a high-fat diet (HFD) to induce obesity by 8 weeks. The animals were divided into 4 \ groups (control, colitis, HFD and colitis + HFD).

**Results:**

Obesity alone did not raise histopathology scores, but the combination of obesity and colitis worsened the scores in the colon compared to colitis group. Despite the reduction in weight gain, there was increased inflammatory infiltrate in both the colon and visceral adipose tissue of colitis + HFD mice due to increased infiltration of macrophages, neutrophils and lymphocytes. Intravital microscopy of VAT microvasculature showed an increase in leukocyte adhesion and rolling and overexpression of adhesion molecules compared to other groups. Moreover, circulating lymphocytes, monocytes and neutrophils in the spleen and cecal lymph nodes were increased in the colitis + HFD group.

**Conclusion:**

Our results demonstrated the relationship between ulcerative colitis and obesity as aggravating factors for each disease, with increased inflammation in the colon and adipose tissue and systemic alterations observed in the spleen, lymph nodes and bloodstream.

## Background

Ulcerative colitis is a disease of incompletely understood etiology and is characterized by inflammation of the colonic mucosa. In its chronic form, inflammation extends into the muscle layer of the colon, with acute (symptomatic) manifestations, asymptomatic periods and relapses after months, years or even decades. Ulcerative colitis inflammation affects the rectum and extends in a retrograde manner, with extensive areas of superficial lesions. In more severe cases, the entire colon can be affected [1,2].

The epidemiology of inflammatory bowel disease suggests that environmental factors such as personal hygiene, smoking and diet contribute to disease onset [3]. Pro-inflammatory markers including IL(interleukin)-6, IL-1 and TNF(tumor necrosis factor)-alpha are increased in colitis. These markers are also increased in obesity that have an inflammatory component [4,5]. Obesity is a multifactorial disease involving endocrine factors, genetics and behavior and directly contributes to systemic inflammation. Several studies have established a relationship between weight gain and levels of inflammatory proteins such as TNF, IL-1, IL-6 and leptin [4,5]. Moreover, studies have suggested that adipokines secreted by adipose tissue (TNFα, IL-1, IL-6, leptin, resistin and adiponectin) are closely associated with inflammatory bowel diseases such as ulcerative colitis [6-13]. In addition, some studies have shown increased pro-inflammatory cytokines in the intestine of obese animals as well as in the adipose tissue of animals with colitis [9,12]. Nonetheless, to the best of our knowledge, inflammatory profile in animals with both diseases was not determined.

The objective of this study was to elucidate the relationship between obesity induced by a hypercaloric, high-fat diet (HFD) and chronic ulcerative colitis induced by intermittent administration of dextran sodium sulfate (DSS).

## Results

As expected, caloric intake was higher and water intake was lower in groups receiving the HFD (Table 1). During DSS administration, both caloric intake and water were reduced in the colitis groups. As a result, weight loss occurred, although body weights returned to normal in the weeks following treatment (data not shown). Colitis was confirmed by rectal bleeding, beginning on the third day after DSS introduction and persisting until one day after its removal. Although caloric intake was similar in both HFD groups, total weight gain was higher only in the HFD group without colitis.

**Table 1:** Parameters of mice from Control and HFD groups receiving chow and high fat diet, respectively or Colitis and Colitis + HFD groups, receiving the respective diets and treated with 2 cycles of DSS (3%) to induce ulcerative colitis

Parameter	Control	Colitis	HFD	Colitis+HFD
Food intake (kcal/mouse)	67.07 ± 3.65^a^	62.47 ± 2.48^a^	112.8 ± 3.92^b^	104.9 ± 4.20^b^
Liquid Intake (mL/mouse)	59.00 ± 3.98^a^	53.28 ± 2.90^a^	39.91 ± 2.35^b^	29.99 ± 0.95^b^
Total weight gain (g)	8.36 ± 0.82^a^	5.84 ± 0.66^a^	10.58 ± 0.81^b^	7.53 ± 0.85^a^
OGTT (A.U.C)^1^	656.9 ± 28.5^a^	668.9 ± 28.5^a^	844.4 ± 27.8^b^	918.2 ± 53.6^b^
AKT mRNA^2 ^(adipose tissue)	0.67 ± 0.23	0.33 ± 0.12	0.30 ± 0.02	0.58 ± 0.14
GLUT4 mRNA^2 ^(adipose tissue)	0.96 ± 0.35	0.50 ± 0.14	0.82 ± 0.33	0.98 ± 0.23

^1^OGTT: oral glucose tolerance test. AUC (area under the curve), ^2 ^mRNA expressed as fold increase compared to control. Results expressed as mean ± standard error. ANOVA + Newman-Keuls post-test, Different letters in a same row = p < 0.05. n = 5 to 7/group.

Lipid profiles (triglycerides, total cholesterol, and LDL and HDL cholesterol) were not affected by diet or colitis (see additional file 1 - Table S1). The glucose tolerance test was altered in both HFD groups. Fasting glycemia. insulinemia, the homeostatic model assessment (HOMA) index and insulin sensitivity were similar among groups (see additional file 1 - Table 1). Despite alterations in glucose tolerance, no changes in expression of serine/threonine protein kinase (AKT/PKS) and glucose transporter type 4 (GLUT4), which are involved in the cascade of insulin signaling and glucose uptake, were detected in adipose tissue (Table 1).

The percentages of blood lymphocytes and monocytes were higher in animals with colitis that received a high-fat diet compared to others (see additional file 2 - Table S2). Other leukocyte subtypes remained unchanged.

### Colon evaluation

**Figure 1:**
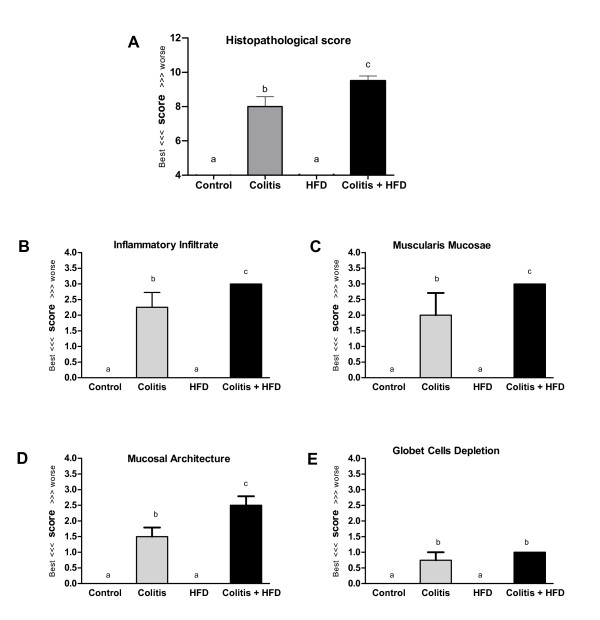
**Histopathological scores (A) and components: inflammatory infiltrate (B), muscularis mucosa thickness (C), mucosa general architecture (D) and goblet cell depletion (E) of mice from control and HFD groups (receiving standard chow or HFD, respectively) or colitis and colitis + HFD groups (receiving the respective diets and treated with 2 cycles of DSS [3%] to induce ulcerative colitis)**. Bars indicate the means, and vertical lines indicate the standard errors. n = 4-5 per group. Statistical tests: One-way ANOVA + Newman-Keuls post-test. Different letters indicate p < 0.05.

Colon histology was observed three weeks after the second DSS cycle. Colons from the HFD and control groups showed intact mucosal architecture and the presence of goblet cells, without signs of congestion or inflammatory infiltrate in the mucosa and submucosa. In animals from the colitis groups, we found goblet cell depletion, irregular thickening of the muscle layer and inflammatory infiltrate, which was constituted mainly of macrophages (data not shown). These findings were more intense in the colitis + HFD group, as determined by histopathological score. The scores for the control and HFD groups were set at zero because no relevant changes were found in mice from either of these groups. However, the damage seen in the colitis group was significantly aggravated by HFD (Figure 1A). When the components of the scores were analyzed separately, a change in mucosal architecture, a thickening of the muscle, inflammatory infiltration and a depletion of goblet cells were also noted in the colitis groups, even after 3 weeks of DSS application, which indicated a chronic condition. The association of colitis with HFD significantly aggravated these changes, except for goblet cell depletion, which was similar in both groups with colitis (Figure 1B-E).

**Figure 2:**
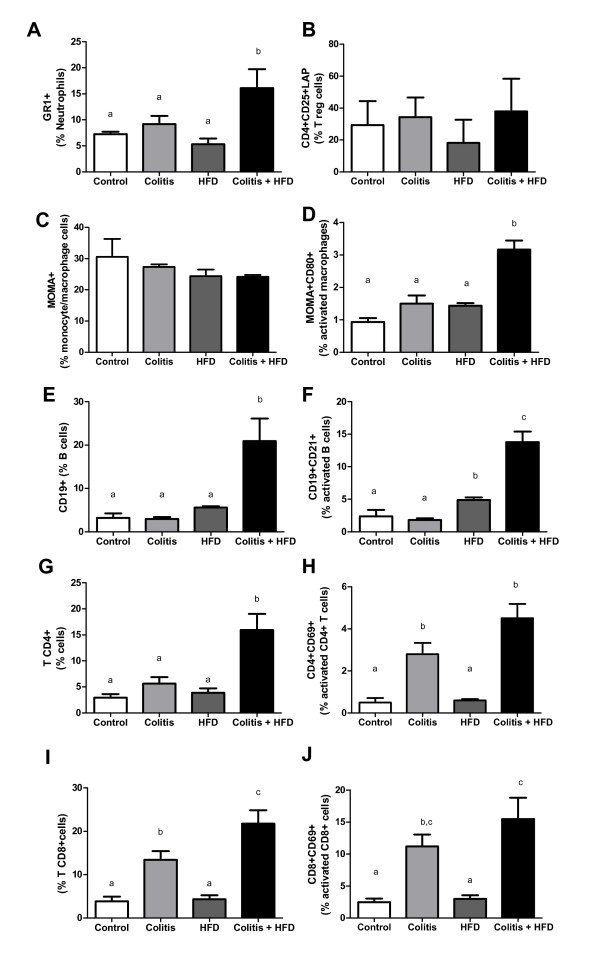
**Percentage of leukocyte subtypes in the colon lamina propria**. Neutrophils (A), regulatory T lymphocytes (B), total macrophages and monocytes (C), activated macrophages (D), B lymphocytes (E), activated B lymphocytes (F), CD4+ T cells (G), activated CD4+ T cells (H), CD8+ T cells (I), activated CD8+ T cells (J) of mice from control and HFD groups (receiving standard chow or HFD, respectively) or colitis and colitis + HFD groups (receiving the respective diets and treated with 2 cycles of DSS [3%] to induce ulcerative colitis). Bars indicate the means, and vertical lines indicate the standard errors. n = 5 per group. Statistical tests: One-way ANOVA + Newman-Keuls post-test. Different letters indicate p < 0.05.

We next investigated the inflammatory infiltrate in the colon. Flow cytometry confirmed that the combination of colitis and HFD increased leukocyte migration and activation in the lamina propria, as seen for neutrophils, T helper (CD4+) cells, B (CD 19+) cells and activated macrophages (MOMA+ CD80+). Activation of CD4+ CD69+ T cells was more related to the presence of colitis, whereas activated B lymphocytes (CD 19+ CD21+) were more frequent in the obese groups (Figure 2). A greater infiltration of total (CD8+) and activated (CD69+ CD8+) T lymphocytes was seen in the colitis group and was exacerbated by HFD (Figure 2).

**Figure 3:**
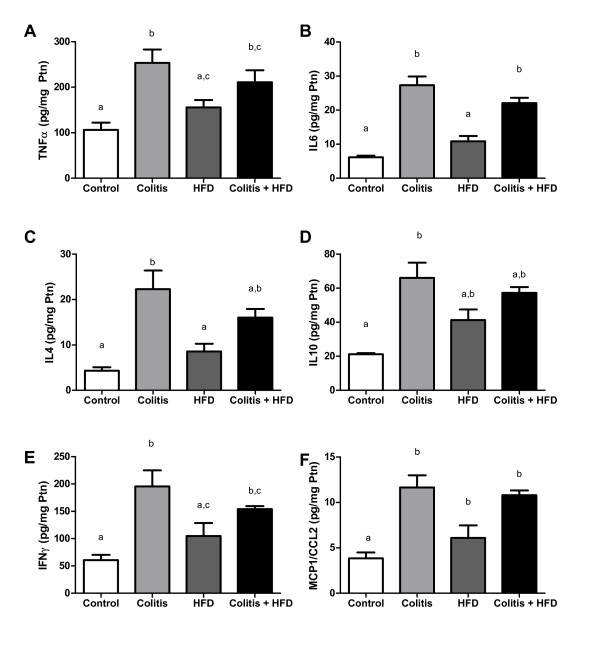
**Lamina propria cytokine and chemokine concentrations**. TNFa (A), IL6 (B), IL4 (C), IL10 (D), IFN-y (E) and MCP1/CCL2 (F) of mice from control and HFD groups (receiving standard chow or HFD, respectively) or colitis and colitis + HFD groups (receiving the respective diets and treated with 2 cycles of DSS [3%] to induce ulcerative colitis). Bars indicate the means, and vertical lines indicate the standard errors. n = 5 per group. Statistical tests: One-way ANOVA + Newman-Keuls post-test. Different letters indicate p < 0.05.

We next assessed colonic production of inflammatory cytokines and chemokines (Figure 3). Mice with colitis, regardless of diet, presented higher concentrations of all measured cytokines (TNF, IL-6, IL-4, IL-10, IFNy), and MCP-1/CCL2. However, the HFD group had cytokine levels that were intermediate to the control colitis groups (Figure 3).

**Figure 4:**
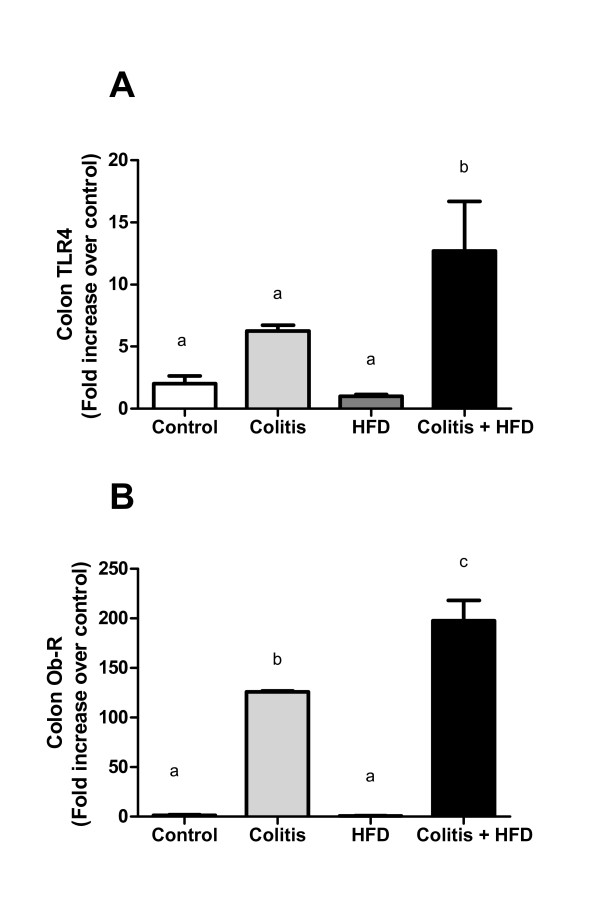
**Expression of TLR4 (A) and Ob-R (B) in the colon mucosa of mice from control and HFD groups (receiving standard chow or HFD, respectively) or colitis and colitis + HFD groups (receiving the respective diets and treated with 2 cycles of DSS [3%] to induce ulcerative colitis)**. Bars indicate the means, and vertical lines the indicate standard errors. n = 5 per group. Statistical tests: One-way ANOVA + Newman-Keuls post-test. Different letters indicate p < 0.05.

The expression of the leptin receptor b (Ob-Rb) and toll like receptor (TLR) 4 showed that the combination of diet and colitis was significantly related to TLR4 overexpression (Figure 4A). Ob-Rb overexpression was seen in the colitis group and was exacerbated in the colitis + HFD group compared to control and HFD groups (Figure 4B).

Changes in the inflammatory profile were less intense in the cecal lymph nodes and spleen, showing only minor alterations (see additional file 2 - Table S2). In the cecal lymph nodes, HFD caused an increase in neutrophils, which was secondary to colitis and the increased activity of T lymphocytes. Interestingly, HFD in the absence of colitis increased the CD4 and CD8 T cell populations. In the spleen, a higher percentage of neutrophils and activated CD4+ T was seen.

### Epididymal adipose tissue

**Figure 5:**
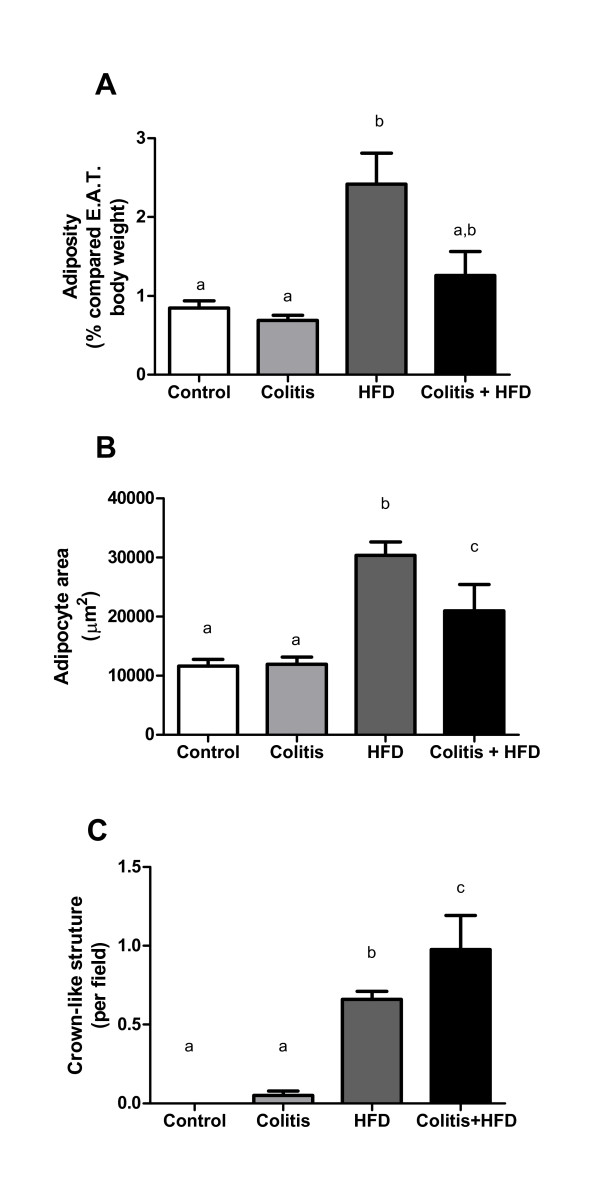
**Percentage of epididymal adipose tissue in relation to body (A), adipocyte area (B) and average of number of crown-like structures (C) of mice from control and HFD groups (receiving standard chow or HFD, respectively) or colitis and colitis + HFD groups (receiving the respective diets and treated with 2 cycles of DSS [3%] to induce ulcerative colitis)**. Bars indicate the means, and vertical lines indicate standard error. n = 5 per group. Statistical tests: One-way ANOVA + Newman-Keuls post-test. Different letters indicate p < 0.05. n = 12-14 per group in (A) and (C) and 4-5 per group in (B).

**Figure 6:**
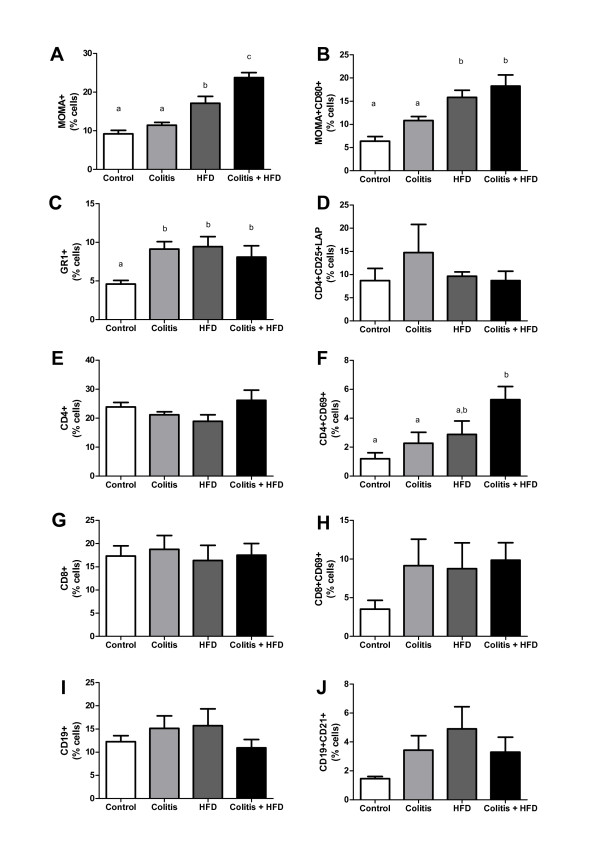
**Macrophages and monocytes (A), activated macrophages (B), neutrophils (C),  regulatory T lymphocytes (D), total (E) and activated (F) CD4+ T cells, total (G) and activated (H) CD8+ T cells, total (I) and activated (J) B cells, activated cytotoxic T lymphocytes (H), total (I) and activated B cells (J) of mice from control and HFD groups (receiving standard chow or HFD, respectively) or colitis and colitis + HFD groups (receiving the respective diets and treated with 2 cycles of DSS [3%] to induce ulcerative colitis). **Bars indicate the means, and vertical lines indicate the standard errors. n = 5 per group. Test: One-way ANOVA + Newman-Keuls post-test. Different indicate p < 0.05.

**Figure 7:**
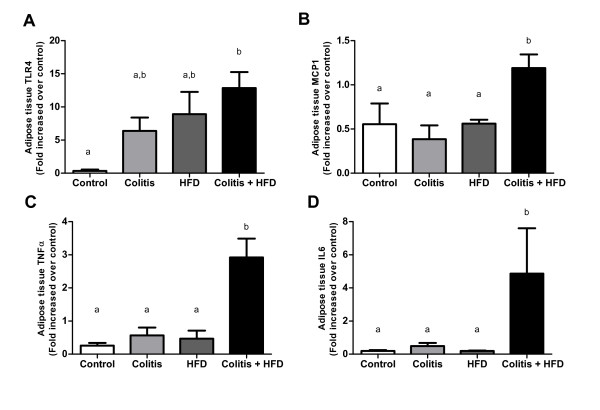
**Expression of TLR4 (A), MCP1/CCL2 (B), TNFα (C) and IL6 (D) in adipose tissue of mice from control and HFD groups (receiving standard chow or HFD, respectively) or colitis and colitis + HFD groups (receiving the respective diets and treated with 2 cycles of DSS [3%] to induce ulcerative colitis)**. Bars indicate the means, and vertical lines indicate the standard errors. n = 5 per group. Statistical tests: One-way ANOVA + Newman-Keuls post-test. Different letters indicate p < 0.05.

HFD mice had a greater expansion of adipose tissue and adipocyte areas that was more intense than in the colitis + HFD group (Figure 5A-B). Despite the reduced adipocyte area, the number of crown-like structures (CLS) was higher in the colitis + HFD mice (Figure 5C), suggesting chronic colitis increased the inflammation in the adipose tissue. In accordance with the CLS results, macrophages (MOMA+) and activated macrophages (MOMA+ CD80+) were increased in the HFD groups (Figure 6A-B) and were significantly higher when HFD was combined with colitis (Figure 6A). Neutrophil (GR1+) infiltration was also increased in all groups compared to the controls, showing that the increase in these cells was secondary to both diet and chronic colitis (Figure 7C). Moreover, more CD4+ T lymphocytes were activated (CD4+ CD69+) in the colitis + HFD animals (Figure 6F). No difference was seen between the groups in relation to the percentage of the other leukocytes (Figure 6D, I -J). Along with the higher number of immune cells in the colitis + HFD group, we found overexpression of TNFα, IL-6 and MCP1/CCL2 (Figure 7B-D) compared to the other three groups. Adipose tissue TLR4 levels were higher in the Colitis + HFD group compared to controls and intermediate to the colitis and HFD groups (Figure 7A).

**Figure 8:**
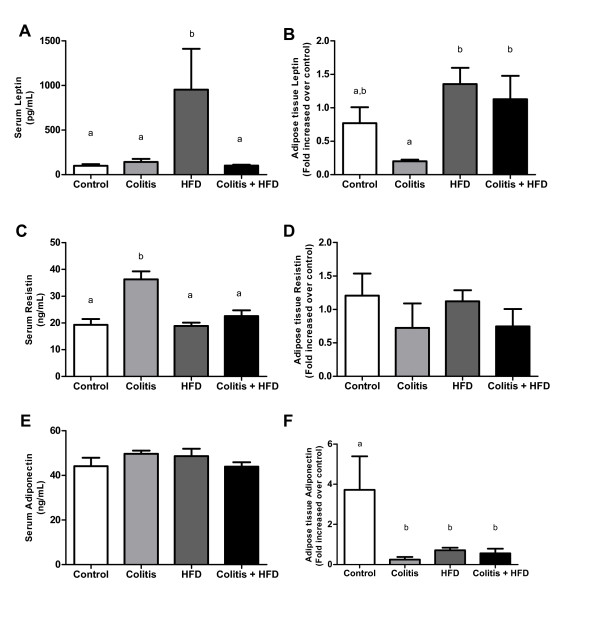
**Serum levels and adipose tissue expression of leptin (A, B), resistin (C,D) and adiponectin (E,F) of mice from control and HFD groups (receiving standard chow or HFD, respectively) or colitis and colitis + HFD groups (receiving the respective diets and treated with 2 cycles of DSS [3%] to induce ulcerative colitis)**. Bars indicate the means, and vertical lines indicate the standard errors. Statistical tests: One-way ANOVA + Newman-Keuls post-test. Different letters indicate p < 0.05. n = 5 per group.

Leptin, resistin and adiponectin interfere with inflammatory responses. Circulating levels of leptin were higher in the HFD group compared with the other three groups (Figure 8A). Leptin expression in adipose tissue was increased in both HFD groups compared to the colitis group (Figure 8B). We observed similar resistin expression in adipose tissue, but resistin serum concentrations were higher in the colitis group compared to the other groups (Figure 8C-D). No changes were observed in serum levels of adiponectin, but its expression was reduced in adipose tissue in all groups compared to control levels (Figure 8E-F).

**Figure 9:**
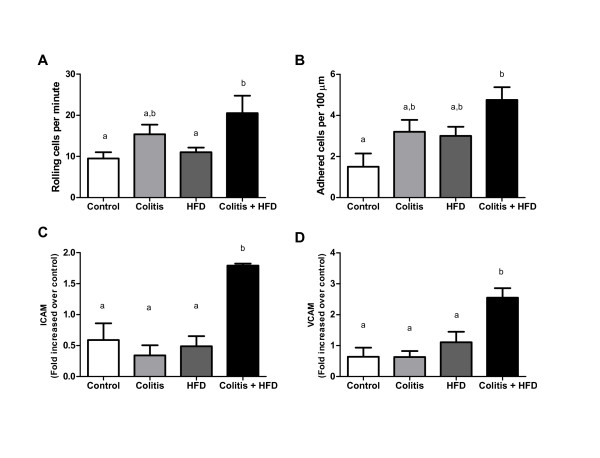
**Intravital microscopy showing the rolling (A) and adhesion (B) of leukocytes on microvasculature of epididymal adipose tissue and ICAM (C) and VCAM-1 (D) expression in control and HFD groups (receiving standard chow or HFD, respectively) or colitis and colitis + HFD groups (receiving the respective diets and treated with 2 cycles of DSS [3%] to induce ulcerative colitis)**. Bars indicate the means, and vertical lines indicate standard error. n = 5 per group. Statistical tests: One-way ANOVA + Newman-Keuls post-test. Different letters indicate p < 0.05.n = 6-8 per group.

The impact of chronic colitis on adipose tissue inflammation was confirmed by intravital microscopy performed in the adipose tissue microvasculature (see additional files 3,4,5,6 - videos 1-4). Leukocyte rolling and adherence and inter-cellular adhesion molecule (ICAM) and vascular cell adhesion protein (VCAM) expression were higher in the colitis + HFD group compared to controls. The remaining groups (colitis and HFD) were maintained at intermediate levels (Figure 9).

## Discussion

Our study shows that HFD prolongs and aggravates the inflammatory manifestations of chronic ulcerative colitis, thereby increasing the pro-inflammatory status of adipose tissue. In our experiment, the analyses were performed 3 weeks after the second cycle of DSS (8^th ^experimental weeks) to permit the evaluation of this relationship out of the acute phase of ulcerative colitis in which metabolic abnormalities are more intense. In the same manner, we obtained a moderate grade of obesity due to the HFD, as seen by the significantly increased weight gain, adiposity and adipocyte area in the HFD group.

As previously described [14], animals provided with HFD consumed less liquid than those fed the standard chow diet. It could be assumed that DSS intake was also reduced in the colitis+HFD group. However, reduced DSS intake was not reflected in the intensity of the disease, as histopathological scores were even more intense in the colitis + HFD group compared to the colitis group.

Colon analyses showed mild inflammation in the colitis group, which became more severe when associated with HFD. Immune dysfunctions have been described both in obesity [15,16] and ulcerative colitis [17]. It is well-known that HFD leads to increased intestinal permeability by several mechanisms, including changes in the expression of tight junction proteins, such as occludins, ZO1 and claudin [18,19]. Thus, we hypothesized that chronic HFD consumption compromises the integrity of the intestinal barrier by disrupting the balance between injury and regeneration of the intestinal epithelium [16,20] and exaggerating the mucosal immune response as result of increased pathogens and antigen entry from the intestinal lumen (including LPS), thereby enhancing the inflammation of ulcerative colitis [20]. TLR4 plays an important role in the intestinal inflammatory response because it is activated either by lipopolysaccharide (LPS) produced by colonic bacteria or saturated fatty acids (FAs), both of which were present in the Colitis + HFD animals. The activation of TLR4 in colon cells could induce the expression and release of pro-inflammatory cytokines [21], as seen in the present study, via activation of NF(nuclear factor)-κB [22], thereby increasing intestinal permeability in ulcerative colitis [17,23]. As a result, luminal antigens may gain access to the lamina propria and trigger naive T cell differentiation, activation and production of inflammatory cytokines. Macrophages in the lamina propria are stimulated by these cytokines and secrete TNFα and IL-6, which enhances local inflammation [24]. Our study supports this sequence of events, as we found increased colonic TLR4 expression, leukocyte (macrophages, lymphocytes and neutrophils) infiltration and inflammatory cytokine release.

The mechanisms of increased intestinal permeability related to obesity are not yet fully understood [25,26]. The persistently high levels of inflammatory cytokines produced by inflamed adipose tissue can cause an imbalance of the intestinal barrier function by altering tight junction proteins [25]. These proteins regulate the selective transport of ions, solutes and peptides from the lumen of the intestine to the bloodstream. Thus, the association between diet-induced obesity and chronic ulcerative colitis enhances transport of pathogens or their derivatives (such as LPS) that were previously restricted by the intestinal barrier to the systemic circulation resulting in inflammatory pathways activation [23,27] in peripheral organ adipose tissue. Interestingly, the changes triggered by chronic colitis were more significant in visceral adipose tissue than in closely related immune organs, such as the spleen and lymph nodes, highlighting the influence of intestinal inflammation on organs that are not traditionally associated with immune responses, such as adipose tissue.

In addition to adipocytes, adipose tissue is composed of stromal vascular cells, monocytes and lymphocytes, which are responsible for immunological balance [28]. Adipose tissue expansion triggers immunological imbalance [15,22] by increasing expression and also activation of TLR-4, which leads to NFκB activation and, consequently, production of pro-inflammatory cytokines [22]. Moreover, the influx of Th1 lymphocytes and macrophages further increases pro-inflammatory cytokine and chemokine production, attracting and activating more macrophages to perpetuate the inflammation [15,22,28]. In our study, the increase in adipose tissue inflammation seen in the Colitis + HFD group was confirmed by the increase in CLS, activated macrophages, lymphocytes and neutrophils and the overexpression of adhesion molecules, TNFα, IL-6, MCP1/CCL2 and TLR4.

This pro-inflammatory scenario was confirmed in vivo by the increased rolling and adhesion of leukocytes in adipose tissue microvasculature.

Although the colitis + HFD group presented lower adiposity and adipocyte area, the number of CLS-forming macrophages was higher compared to that of other groups. CLS represents the conclusion of the relatively long life of adipocytes and is related to the macrophage response to changes in adipose tissue that lead to adipocyte death or apoptosis [29]. In addition to the CLS, the increase in activated CD4+ T cells in colitis + HFD mice leads to increased MCP-1/CCL2 release in this tissue [15], which induces migration and activation of macrophages that originate from the CLS and enhance inflammation [29].

Although it is a classical inflammatory marker, we observed an increase in serum resistin in the colitis group that was not observed in the Colitis + HFD group. Increased serum resistin in patients with ulcerative colitis has been previously described [7,8,13] and is an early marker of inflammation [8]. Resistin is also produced by peripheral blood mononuclear cells and macrophages [30]. Therefore, serum levels were not correlated to resistin expression in epididymal adipose tissue. The absence of increased resistin in the HFD group disagrees with previous literature [31]. This discrepancy may be due to the duration of the present experiment and the moderate obesity observed, which were not sufficient to induce changes in this parameter.

Although circulating levels of adiponectin were similar among groups, its expression in adipose tissue was reduced in all groups compared to the control group. This fact has been previously described in animals fed HFD [32], but its pathological significance in inflammatory bowel disease is still controversial. Human studies have shown both increased [7,33] and decreased [13,34] serum adiponectin levels in patients with ulcerative colitis. Studies in mice are even more controversial. Two studies using adiponectin knockout mice with DSS-induced ulcerative colitis showed opposite results. One found a protective effect of adiponectin, as the knockout animals displayed a more severe ulcerative colitis [10], while the other study showed a protective effect of adiponectin deletion against the development of ulcerative colitis [6]. Pro-inflammatory factors may decrease the expression of adiponectin [35], which would explain its decreased expression in visceral adipose tissue. Moreover, subcutaneous fat, rather than visceral fat (as measured in this study), is the main site of adiponectin production.

Leptin is produced in adipose tissue in response to fat mass and controls appetite and energy expenditure by hypothalamic pathways [36]. Leptin also modulates several immune and inflammatory responses, such as monocyte and T lymphocyte activation, phagocytosis and TNFa, IL-6, and IFN-y production by peripheral blood mononuclear cells [36-38]. In our study, leptin expression was increased in adipose tissue in both HFD groups compared to the colitis group. However, the higher circulating leptin concentration was seen only in the HFD group and not the Colitis + HFD group. This may be due to increased leptin/Ob-R receptor binding because the receptor is overexpressed in inflamed colons of colitis animals [39,40]. Therefore, we believe that leptin/receptor interactions in the colons of the colitis + HDF animals are partially responsible for maintaining local colonic inflammation.

A previous study [29] has reported increased leptin expression in the colons of mice with colon cancer that was secondary to DSS-induced ulcerative colitis. As in our work, the authors observed increased leptin expression in the fatty tissue of animals with colitis associated with HFD. These results agree with ours and reinforce the association between leptin and inflammation.

To the best of our knowledge, this is the first time that crosstalk between inflammatory components of ulcerative colitis and obesity has been shown to lead to mutual exacerbation. We believe that this relationship is supported by increased leptin uptake due to Ob-Rb overexpression induced by colitis. Leptin then activates immune cells by producing IL-6, IL-1p and chemokine (C-X-C motif) ligand 1 CXCL1[41] and supports intestinal inflammation caused by chronic ulcerative colitis. However, changes in intestinal permeability triggered by colitis (and aggravated by HFD) allow the greater input of LPS and fatty acids in the systemic circulation, which bind to TLR4 receptors on the surface of adipocytes and macrophages in the expanded adipose tissue, thereby activating NF-ĸB signaling and reinforcing the systemic inflammation.

## Conclusion

In conclusion, HFD during chronic ulcerative colitis creates an environment of reciprocal activation and worsens inflammation in adipose tissue and colon.

## Methods

The project was approved by the Ethics Committee for Animal Experimentation at the Federal University of Minas Gerais (CETEA/UFMG # 110/2010).

Six-week-old male C57BL/6 mice were group-housed in an environment with light cycles of 12 hours (7:00 to 19:00) and controlled temperature (20 to 24°C). All mice had free access to food and water for 8 weeks.

The animals were divided into the following groups: control, colitis, HFD and colitis + HFD. Animals from the control and colitis groups were fed a standard chow diet (Labina ^®^, 7.8% energy as fat, 50.3% in the form of carbohydrates, 41.9% protein, caloric density of 2.18 kcal/g) for 8 weeks, while mice from the HFD and Colitis + HFD groups received a high-fat diet (HFD) to induce obesity [42]. The composition of the HFD was 61% energy as fat, 24.5% carbohydrates, and 14.5% protein and had a caloric density of 5.21 kcal/g.

Chronic ulcerative colitis was induced in the colitis and colitis + HFD groups by replacing drinking water with a solution of 3% (w/v) dextran sulfate sodium (DSS, 36.000 to 50.000, MP Biomedicals) for 5 days as previously described [9,43] at the 1^st ^and 4^th ^experimental weeks. The animals without induction of colitis (the control and HFD groups) received water throughout the experiment. At the end of the 8^th ^experimental week, all animals were euthanized under anesthesia after overnight fast. Blood, adipose tissue, spleens, lymph nodes and colons were removed for analysis.

### Blood analysis

Samples were collected without anticoagulant for lipid profiling and insulinemia analysis and with EDTA/KF for glycemia analysis. Blood glucose, total and HDL cholesterol and triglycerides were determined as previously described [44] using commercial kits (Labtest, Brazil). Non-HDL cholesterol was calculated as the difference between total cholesterol and HDL cholesterol. Insulin was measured by a radioimmunoassay kit (Millipore, USA), according to the manufacturer's instructions for the calculation of HOMA-IR and HOMA-BETA [45]. Oral glucose tolerance and insulin sensitivity tests were performed 1 and 3 days before sacrifice, respectively, as previously described [46]. The total and differential count of leukocytes were performed the day before sacrifice as described by Nowakowski et al. [47].

### Histological Analysis

Colon tissue was removed and fixed in 10% formalin for 4 hours. The visceral (epididymal) adipose tissue (VAT) was removed, washed in saline solution and fixed in 10% formalin. The colon and adipose tissue were embedded in paraffin and processed into 10um histological sections and stained with hematoxylin and eosin [48]. The sections were analyzed using an optical microscope coupled to a camera to capture images with a 100x magnification and analyzed with Image Pro Plus (Media Cybernetics, MD, USA). Semi-quantitative scoring for the colon was performed as previously described [49]. The adipocyte area was calculated by the analysis of 100 adipocytes/section/per animal. The crown-like structures (CLS) were determined by the average of the number of CLS in 10 fields per mouse.

### Flow Cytometry

The leukocytes in colonic lamina propria were separated as previously described [50,51]. Cell suspensions were incubated with appropriate antibodies (against CD4, CD8, CD69, CD25, LAP, CD21, CD19, MOMA, CD80, or GR1), fixed with formaldehyde and read in a flow cytometer (FACScan - Becton Dickinson, USA) using the CELLQuestTM program (USA). Analyses were performed with FlowJo software 7.6 (USA) in the specific quadrants for each cell type.

### Cytokines and chemokines by ELISA

We analyzed TNFa, IFNy, IL-4, IL-6, IL-10 and MCP1/CCL2 expression in the colon. Serum leptin, adiponectin and resistin were measured by ELISA. Colons were washed with PBS, dried, weighed and homogenized with cytokine extraction solution (BSA 0.05%, aprotinin 0.02 mg/mL, benzethonium chloride 0.05 mg/mL, NaCl 0.023 mg/mL, EDTA 0.37 mg/mL, PMSF 0.02 mg/mL, 0.5 mL Tween20/PBS 1x mL) and centrifuged (10,000rpm, 10 min at 4°C). The supernatant was used for ELISA analysis, according to the manufacturer's protocol (R & D Systems).

### Reverse transcription polymerase chain reaction (RT-PCR)

The epididymal adipose tissue was removed for evaluation of TNFa, IL-6, monocyte chemotactic protein-1 (MCP1/CCL2), serine/threonine protein kinase (AKT/PKS), glucose transporter type 4 (GLUT4), intercellular adhesion molecule 1 (ICAM-1), vascular cell adhesion protein 1 (VCAM 1), toll-like receptor 4 (TLR4), resistin, adiponectin and leptin expression. RT-PCR was also performed in colon tissue to evaluate expression of TLR4 and leptin receptor b (Ob-Rb). Samples were transferred to Trizol solution (Invitrogen, USA) for RNA extraction as described previously [52]. In animals receiving DSS, the extracted colon RNA was purified with an RNA purification kit (Qiagen, Germany), according to the manufacturer's instructions. To obtain cDNA, samples were placed in the thermocycler at 72°C for 5 min for annealing and 42°C for 3 h and 72°C for 15 min for transcription. Real-time semi-quantitative PCR was performed using Power SYBR MasterMix (AppliedBiosystems, Foster City, California, USA) and specific primers (Table 2) in an ABI 7900 HT Fast Real-Time PCR System (Applied Biosystems). PCR reactions were initiated with a 2 minute incubation at 95 °C, followed by 35 cycles at 95 °C for 15 seconds and at 60°C for 60 seconds. The results were normalized to β-actin expression and are expressed as the fold increase compared to the control [52].

**Table 2:** List of used primers

	Foward	Reverse
**Leptina**	5CCTGTCGCTTTGGTCCTATCTG3'	5'AGGCAAGCTGGTGAGGATCTG3'
**Adiponectina**	5'AGGTTGGATGGCAGGC3'	5'GTCTCACCCTTAGGACCAAGAA3'
**Resistina**	5'AGACTGCTGTGCCTTCTGGG3'	5'CCCTCCTTTTCCTTTTCTTCCTTG3'
**TNFα**	5'CGTCGTAGCAAACCACCAAG3'	5'GAGATAGCAAATCGGCTGACG3'
**IL6**	5'ACAACCACGGCCTTCCCTACTT3'	5'CACGATTTCCCAGAGAACATGTG3'
**MCP1**	5'CCACTCACCTGCTGCTACTACT3'	5'TGGTGATCCTCTAGCTCTCC3'
**GLUT4**	5'CTGCAAAGC GTAGGTACCAA3'	5'CCTCCCGCCCTTAGTTG3'
**AKT**	5'GGCAGGAAGAAGAGACGATGG3'	5'CCATCTCTTCAGCCCCTGAG3'
**VCAM**	5'CCTCACTTGCAGCACTACGGGC3'	5'TTTTCCAATATCCTCAATGACGGG3'
**ICAM**	5'TGCGTTTTGGAGCTAGCGGACCA3'	5'CGAGGACCATACAGCAGCTGCAG3'
**TLR4**	5'TGACAGGAAACCCTATCCAGAGT3	5'TCTCCACAGCCACCAGATTCT3'
**Ob-Rb**	5'GTG TGA GCA TCT CTC CTG GAG3'	5 ACC ACA CCA GAC CCT GAA AG3'
**β-actina**	5'CTGCCTGACCAAGTC3'	5'CAAGAAGGAAGGCTGGAAAGG A3'

### Intravital microscopy

Intravital microscopy was performed in the epididymal adipose tissue and colon microvasculature. Mice were anesthetized with 10 mg/kg xylazine and 100 mg/kg ketamine hydrochloride, injected i.p. The right jugular vein was cannulated, and rhodamine 6G (Sigma, St. Louis, MO, USA) was injected intravenously (i.v.; 0.15 mg/kg) to visualize the leukocyte/endothelial cell interactions. Rhodamine epi-illumination was achieved with a 150 W variable HBO mercury lamp in conjunction with a Zeiss filter set 15 (546/12 nm band-pass filter, 580 nm Fourier transforms, 590 nm late potentials; Zeiss, Wetzlar, Germany). Microscopic images were captured using a Nikon Eclipse 50i (Nikon Instruments Inc., Japan) microscope (x20 objective) with a video camera (5100 HS; Panasonic, Secaucus, NJ) and consecutive digital recordings using both filters. Data analysis was performed off-line. Rolling leukocytes were defined as those cells moving slower than the cells at a regular flux in a given vessel. The flux of rolling cells was measured as the number of rolling cells passing by a given point in the venule per minute, with results expressed as cells/minute. A leukocyte was considered to be adherent if it remained stationary for at least 30 s, and total leukocyte adhesion was quantified as the number of adherent cells within a 100 μm length of venule, with results expressed as cells/100 μm [53].

### Statistical Analysis

The results were evaluated for normal distribution by the Kolmogorov-Smirnov test, and outliers were identified by the Grubbs and Box-Plot tests. A one-way ANOVA followed by a Newman-Keuls multiple comparison test was used for normally distributed data, and the Kruskal-Wallis test followed by Dunn's multiple comparison test were used for non-parametric data. The results are expressed as the mean ± standard error. Statistical analysis was performed using GraphPad Prism 5.0 software, (USA) with a significance level of 5% (p < 0.05).

## List of Abbreviations

AKT/PKS: serine/threonine protein kinase; BSA: bovine serum albumin; CD: cluster of differentiation; CLS: crown-like structure; CXCL1: chemokine (C-X-C motif) ligand 1; DSS: dextran sodium sulfate; EDTA/KF: Ethylenediaminetetraacetic acid/potassium fluoride; ELISA: Enzyme-Linked Immunoabsorbent Assay; GLUT4: glucose transporter type 4; HDL: high density lipoproteins; HFD: high-fat diet; HOMA: homeostatic model assessment; ICAM: inter-cellular adhesion molecule 1; IFN: interferon; IL: interleukin; IR: insulin resistance; Kcal/g: kilocalories per gram; LDL: low density lipoproteins; LPS: lipopolysacharide; MCP1/CCL2: monocyte chemotatic protein; RT-PCR: Reverse transcription polymerase chain reaction; NaCl: sodium chloride; NF-ĸB: nuclear factor ĸB; Ob-Rb: leptin receptor b; PMSF: phenylmethanesulfonylfluoride; TLR4: receptor like toll 4; TNF: tumor necrosis factor; VAT: visceral adipose tissue; VCAM: vascular cell adhesion protein

## Competing interests

The authors declare that they have no competing interests.

## Authors' contributions

LGT designed and conducted research, analyzed data and wrote the paper. AJL and ECA helped in conducting research. ACA and AMCF helped in flow cytometry and citokynes ELISA. NVB and DCCM carried out intravital microscopy and helped in histological analysis. CCC carried out the radioimmunoassay. AVMF carried out the adipokines ELISA. JIAL supervised the work and wrote the paper. All authors read and approved the final manuscript.

## Supplementary Material

Additional file 1 **Parameters of lipid profile, glucose homeostasis, intravital microscopy of the colonic microvasculature**. Parameters of lipid profile, glucose homeostasis, intravital microscopy of the colonic microvasculature (rolling and adherent cells) of control and HFD groups (receiving standard chow or HFD, respectively) or colitis and colitis + HFD groups (receiving the respective diets and treated with 2 cycles of DSS [3%] to induce ulcerative colitis). Click here for file

Additional file 2 **Profile of immune cells in blood, spleen and lymph node**. Profile of immune cells in blood, spleen and lymph node of control and HFD groups (receiving standard chow or HFD, respectively) or colitis and colitis + HFD groups (receiving the respective diets and treated with 2 cycles of DSS [3%] to induce ulcerative colitis). Click here for file

Additional file 3 **Intravital microscopy in control adipose tissue**. Intravital microscopy performed in adipose tissue microvasculature of group Control. Click here for file

Additional file 4 **Intravital microscopy in colitis adipose tissue**. Intravital microscopy performed in adipose tissue microvasculature of group Colitis. Click here for file

Additional file 5 **Intravital microscopy in HFD adipose tissue**. Intravital microscopy performed in adipose tissue microvasculature of group HFD. Click here for file

Additional file 6 **Intravital microscopy in colitis + HFD adipose tissue**. Intravital microscopy performed in adipose tissue microvasculature of group colitis + HFD, showing an increase of rolling and adherent leukocyte compared to control. Click here for file
