# Supplementary material for: The combination of high-fat diet-induced obesity and chronic ulcerative colitis reciprocally exacerbates adipose tissue and colon inflammation
Source: Lipids Health Dis. 2011 Nov 10;10:204. doi: 10.1186/1476-511X-10-204 (PMC3254137; doi:10.1186/1476-511X-10-204)
Supplement: Additional file 1 — Parameters of lipid profile, glucose homeostasis, intravital microscopy of the colonic microvasculature. Parameters of lipid profile, glucose homeostasis, intravital microscopy of the colonic microvasculature (rolling and adherent cells) of control and HFD groups (receiving standard chow or HFD, respectively) or colitis and colitis + HFD groups (receiving the respective diets and treated with 2 cycles of DSS [3%] to induce ulcerative colitis). [file 1476-511X-10-204-S1.PDF]

Table S1: Parameters of lipid profile, glucose homeostasis, intravital microscopy of the colonic microvasculature (rolling and adherent cells) of control and HFD groups (receiving standard chow or HFD, respectively) or colitis and colitis + HFD groups (receiving the respective diets and treated with 2 cycles of DSS [3%] to induce ulcerative colitis).

	**Control**	**Colitis**	**HFD**	**HFD + Colitis**
Triglycerides (mM)	0.86±0.09	0.69±0.08	0.56±0.06	0.64±0.11
Total cholesterol (mM)	2.50±0.11	2.30±0.09	2.52±0,20	2.35±0,36
HDL cholesterol (mM)	0.91±0.05	0.75±0.13	0.85±0.08	0.75±0.10
NonHDL cholesterol (mM)	1.59±0.13	1.55±0.14	1.67±0.16	1.61±0.31
Glycemia (mM)	7.54±0.62	8.77±0.25	6.96±0.27	6.47±0.76
Insulinemia (pM)	13.40±6.20	21.35±5.48	27.55±8.021	37.36±26.22
HOMA-IR	0.008±0.004	0.01±0.002	0.012±0.004	0.019±0.015
HOMA-beta	0.07±0.02	0.34±0.22	0.21±0.07	0.32±0.07
Insulin Sensitive (AUC)[#]	279.7±20.03	304.8±10.79	328.5±22.54	292.5±30.2
Cell adhesion[&]	2.8 + 0.7	3.3 + 0.6	1.8 + 0.9	2.8 + 0.5
Rolling cells*	20.4 + 5.1	24.1 + 4.7	25.0 + 8,2	26.0 + 2.4

The results are expressed as the mean ± SEM, n=10/6/10/8 for lipid profile and glucose homeostasis. Statistical tests: One-way ANOVA and post-test Newman-Keuls; $p < 0.05$. [#] Insulin Sensitive test, area under the curve (AUC), [&]Colon microvasculature cell adhesion (cell/100µm) and *rolling cells (cell/min) n=5/group for rolling and adherent cells.
