# Supplementary material for: The combination of high-fat diet-induced obesity and chronic ulcerative colitis reciprocally exacerbates adipose tissue and colon inflammation
Source: Lipids Health Dis. 2011 Nov 10;10:204. doi: 10.1186/1476-511X-10-204 (PMC3254137; doi:10.1186/1476-511X-10-204)
Supplement: Additional file 2 — Profile of immune cells in blood, spleen and lymph node. Profile of immune cells in blood, spleen and lymph node of control and HFD groups (receiving standard chow or HFD, respectively) or colitis and colitis + HFD groups (receiving the respective diets and treated with 2 cycles of DSS [3%] to induce ulcerative colitis). [file 1476-511X-10-204-S2.PDF]

Table S2: Profile of immune cells in blood, spleen and lymph node of control and HFD groups (receiving standard chow or HFD, respectively) or colitis and colitis + HFD groups (receiving the respective diets and treated with 2 cycles of DSS [3%] to induce ulcerative colitis).

	Control	Colitis	HFD	HFD + Colitis
**BLOOD**				
**Total number of cells**	4138±205	5405±799	4059±339	4449±441.6
**Neutrophils (%)**	42.3±6.11	50.33±6.59	33.43±3.05	29.88±5.50
**Eosinophils (%)**	1.3±0.42	2.00±0.52	3.43±0.97	1.25±0.53
**Monocytes (%)**	1.3±0.61[a]	1.67±0.33[a]	2.80±0.20[ab]	4.62±0.60[b]
**Basophils (%)**	9.8±1.58	5.00±1.37	9.43±2.44	6.25±1.06
**Lymphocytes (%)**	38.2±3.82[a]	41.00±5.59[a]	50.71±2.88[a]	62.86±3.34[b]
**CECAL LYMPH NODE**				
**T helper (%)**	30.83±2.87[a]	35.69±1.78[ab]	39.85±3.11[b]	27.33±1.59[a]
**Activate T helper cells (%)**	3.79±0.36[ab]	3.77±0.47[ab]	4.49±0.43[a]	2.93±0.07[b]
**Cytotoxic T cells (%)**	19.22±2.62[a]	18.03±1.76[a]	27.57±2.89[b]	15.05±0.53[a]
**T reg cells (%)**	0.55±0.09[a]	0.34±0.04[b]	0.22±0.04[b]	0.17±0.04[b]
**B cells (%)**	33.36±1.78	40.87±5.55	26.63±6.06	41.45±3.49
**Monocytes/macrophages (%)**	57.72±1.87[a]	62.14±4.92[a]	25.00±6.83[b]	60.42±4.11[a]
**Neutrophils (%)**	3.10±0.45[a]	7.33±1.16[b]	4.05±0.44[a]	10.76±1.73[c]
**SPLEEN**				
**T helper (%)**	23.3±0.9	25.10±2.21	20.37±2.66	20.05±2.86
**Cytotoxic T cells (%)**	14.3±0.5[a]	13.64±0.95	13.12±2.23	11.72±2.12
**T reg cells (%)**	0.6±0.9	0.59±0.19	0.48±0.07	1.17±0.24
**B cells (%)**	58.1±2.5	50.46±1.19	51.61±0.28	47.34±4.76
**Monocytes/macrophages (%)**	26.2±1.4	23.22±2.90	26.42±1.81	24.24±3.11
**Activated macrophages (%)**	11.2±1.6	11.74±1.41	11.20±1.01	11.69±2.08
**Neutrophils (%)**	4.91±0.54[a]	7.81±2.65[a]	7.81±1.57[a]	26.46±6.04[b]

The results are expressed as the mean ± SEM. ANOVA + Newman-Keuls post-test. $p<0.05$.
